# Factors affecting the genetic diversity of *Lotus corniculatus* in the Hemi-boreal zone of Baltic States and their agronomical implications

**DOI:** 10.3389/fpls.2026.1748495

**Published:** 2026-02-04

**Authors:** Yaqoob Sultan, Izhar Ullah, Peter Paľove-Balang, Asif Mukhtiar, Muhammad Mudasir, Michaela Bačovčinová, Vilma Kemešyte, Aurelija Liatukiene, Giedrius Petrauskas, Eglė Norkevičienė

**Affiliations:** 1 Department of Grass Breeding, Institute of Agriculture, Lithuanian Research Centre for Agriculture and Forestry, Akademija, Lithuania; 2 Department of Horticulture, Faculty of Agriculture, Ondokuz Mayıs University, Samsun, Türkiye; 3 Institute of Biology and Ecology, Faculty of Science, P.J. Šafárik Unversity in Košice, Košice, Slovakia; 4 Department of Botany, University of Agriculture Faisalabad, Faisalabad, Punjab, Pakistan; 5 Department of Crop Sciences and Agroforestry, Faculty of Tropical AgriSciences, Czech University of Life Sciences Prague, Prague, Czechia

**Keywords:** Baltic States, biodiversity conservation, genetic diversity, Hemi-boreal zone, *L. corniculatus*, molecular markers

## Abstract

Bird’s-foot trefoil (BFT) is an underutilized perennial legume of increasing importance for sustainable agriculture in Europe, particularly within the Hemi-boreal zone of the Baltic states. It is a resistant, symbiosis-forming, and abiotic stress resistance making it a nitrogen-fixing soil, high potential of biodiversity conservation, pasture restoration, and low input farming systems. Yet, molecular genetic investigations of BFT with systematic investigations populations, of Lithuania, of Latvia, and of Estonia, are practically non-existent, to develop a critical knowledge gap in the agricultural development of the region and conservation planning. This gap is filled by this review through (1) synthesizing global evidence on BFT genetic diversity, population structure, adaptive traits, and characteristics revealed by molecular markers; (2) surveying the little information on the same already available distribution, habitat diversity, and ecotypic variation of the Baltic region; (3) the critical assessment of the applicability of the findings of neighboring European, Transcaucasian, and Central Asian races to Baltic Hemi-boreal states; and (4) setting out a research framework and future research priorities in Baltics-specific genetic studies. We point out the action of ecological gradients, climatic anthropogenic activities, pressures, and biotic interactions on population differentiation and adaptability based on research of the neighboring lands and ecological zones. By directly filling in the existing gap of lack of Baltic-specific molecular data, our analysis creates a level of cognition, which is a synthesis of global comprehension research and a knowledge road map of addressing gaps of knowledge that are critical. The findings underscore that BFT needs genetic diversity to be able to persist despite alteration. Hemi-boreal status and satisfaction of European Union biodiversity and agriculture sustainability goals. Local genetic resources will be tapped through the collection of customized germplasm, which will be molecularly characterized and bred according to their specific ecotype. This approach is essential for developing robust forage systems and supporting productive grassland restoration in the Baltic States. The findings underscore that genetic diversity in BFT is essential for its persistence under changing Hemi-boreal conditions and for meeting European Union biodiversity and agriculture sustainability goals. High genetic diversity provides the adaptive foundation necessary for breeding stress-tolerant cultivars, enhancing nitrogen fixation efficiency, and maintaining ecosystem resilience under climate variability and evolving agricultural practices. The conservation of local genetic resources, molecular characterization, and breeding of ecotypes will be crucial for utilizing these resources to develop resilient forage systems and promote efficient grassland recovery in the Baltic States, where baseline genetic diversity data remains limited.

## 1 Introduction

The importance of a comprehensive investigation of legume crops has become even more urgent, as they are nestled away inside the strategic frameworks of the European Green Deal, the Farm to Fork plan, the EU biodiversity plan for 2030, and the ambitious EU Zero Pollution objective (Mihalczo, 2024). The implementation of these comprehensive policies will only be effective if agricultural methods are established that are both environmentally friendly and commercially competitive. Legume crops emerge as pivotal agents capable of diversifying the prevailing simplified rotations dominating Europe, thereby enhancing the sustainability of European farming systems (Notz et al., 2023). In recent decades, the once-overlooked BFT has emerged as a rediscovered resource for sustainable agriculture in Europe (King, 2023). Traditionally, this group of plants classified as minor legumes has been marginalized in comparison to its main counterparts in terms of scientific effort and attention. According to (Ruiz et al., 2024), the four most significant species in the *Lotus* genus are *L. corniculatus*, *L. uliginosus*, *L. tenuis*, and *L. subbiflorus* from an agronomic perspective. Likewise, *L. uliginosus* Schkuhr (syn. *L. pedunculatus* Cav., Greater Bird’s-foot-trefoil) shows taxonomic uncertainty, with some authors treating both as synonyms and others as distinct species (Sell and Murrell, 2009). As we navigate the evolving landscape of agricultural priorities and sustainability, the spotlight on legume diversity and promoting innovative technologies for growing them becomes vital for shaping the future of European agriculture. It is underutilised species with high potential for novel cropping systems and markers (Xavier, 2023).

Genetic diversity of BFT may be of value in a Hemi-boreal environment, the ecological zone between boreal forest and temperate forest (Petrokas and Manton, 2023). The conditions of this region are described by a unique combination of environmental factors, and the genetic diversity of BFT might be the key to its adaptation to different environmental conditions. Maintaining a diverse gene pool enables the species to better cope with the Hemi-boreal environment, including changing temperatures and weather extremes (Petrokas and Manton, 2023).

In Lithuania, semi-natural grasslands and meadows that support BFT have undergone substantial decline and land-use conversion in recent decades, largely due to agricultural intensification (Juknelienė et al., 2021; Povilaitis et al., 2014). Between 2012 and 2014, a comprehensive survey mapped 77,189 hectares of meadows and EU-protected grassland habitats (Juknelienė et al., 2021). Within only four years, 9% of these habitats had been altered or lost, being replaced by crop fields, mixed plantations, and cultivated forests. Furthermore, 24% of permanent pastures and grasslands, natural grasslands, and wetlands showed continued decline from 2018 to 2022, while arable land expanded by 8% (Povilaitis et al., 2014).

The forthcoming measures to implement the EU Biodiversity Strategy for 2030 will exert significant influence on agriculture and the management of permanent grasslands across Europe. Strategies targeting groundwater protection, fertilizer reduction, and insect conservation will impose additional pressures on grassland management and species composition (Eickmeyer, 2021). Halting the loss of semi-natural permanent pastures and grasslands is therefore a key action for stopping biodiversity decline in Europe (Eickmeyer, 2021). European Union regulations mandate the restoration of perennial meadows and pastures when their area declines beyond allowable limits (Commission Delegated Regulation (EU) 2022/126).

BFT has superior tolerance to waterlogging, acidic soils, and moderate drought compared to white clover, making it an extremely flexible forage legume in low-input agricultural systems (Nguyen, 2022). Its agronomic value is further enhanced by high forage quality and the presence of condensed tannins, which improve protein utilization by reducing ruminal protein degradation and preventing bloat in grazing ruminants—an important advantage over alfalfa and conventional clovers (Kelln et al., 2020). The species demonstrates exceptional persistence under continuous grazing and maintains nutritional value across multiple harvests (Bostan et al., 2025). In addition, BFT forms highly specific and efficient symbiotic associations with nitrogen-fixing rhizobia, including *Bradyrhizobium* sp., *Rhizobium* sp., *Ensifer* sp., and *Mesorhizobium loti*, thereby contributing to soil nitrogen enrichment and reducing reliance on synthetic fertilizers (Sujkowska-Rybkowska et al., 2020). Such symbiotic specificity indicates that genetic variation in the plant host can influence nitrogen fixation efficiency and stress tolerance.

This effectiveness is ecosystem-dependent, and the natural rhizobia populations associated with native legumes, particularly in ultramafic soils, remain poorly characterized. The species predominantly occurs in open grassland habitats and is found in a wide range of well-drained environments, including meadows, wastelands, and roadside verges. It colonizes diverse soil types, from dry and nutrient-poor to wet and fertile substrates, allowing establishment in various ecosystems across Europe (Drobná, 2010). The ecological breadth observed across Europe suggests underlying genetic differentiation driven by local adaptation processes.

The selection of the well-wintering, long-lived variety ‘Gelsvis’ was achieved in the Hemi-boreal zone of Lithuania in the 1950s through interspecific hybridization and individual selection (Bobinas, 2013). Although ‘Gelsvis’ is considered an old landrace, no cultivars in Lithuania have been developed through direct selection from locally adapted populations, despite their potential suitability to local climatic and soil conditions. Genetic diversity represents the raw material for adaptive evolution and crop improvement. Populations with high genetic diversity harbor greater allelic variation that can be exploited for breeding cultivars with enhanced stress tolerance, improved forage quality, and efficient symbiotic nitrogen fixation (Mishra, 2025). Conversely, populations with low genetic diversity may exhibit reduced adaptive capacity and increased vulnerability to environmental stresses, pests, and diseases (Mukhtar & Arif, 2024). Understanding the genetic diversity and structure of BFT within specific regions enables identification of populations containing unique adaptive alleles; informed germplasm collection strategies for conservation and breeding; prediction of population responses to climate change; and development of region-specific cultivars that maintain ecosystem services while meeting production goals (Delrot et al., 2020).

The genus *Lotus* exhibits high morphological, ecological, and genetic diversity worldwide (Abraham et al., 2015). Genetic diversity can be assessed using morphological, biochemical, and molecular markers, with ISSR markers commonly employed for preliminary biodiversity assessments (Merkouropoulos et al., 2017). Restoration of seminatural habitats in the Hemi-boreal zone requires the use of regional plant genetic material; however, regional seed multiplication systems and scientific data on seed traits of local legume species remain insufficient. Despite the ecological and agronomic importance of BFT in the Baltic States, a comprehensive literature review reveals a critical gap: no published studies have systematically characterized the genetic diversity of Lithuanian, Latvian, or Estonian BFT populations using molecular markers. This absence stands in stark contrast to the extensive genetic research conducted on populations from Transcaucasia, Mediterranean regions, and Central/Western Europe. The extent of the Baltic data is void and no DNA-based molecular marker studies (AFLP, SSR, SNP, ISSR) have been conducted on Baltic populations; no population genetic structure analysis specific to the Baltic region exists; no molecular characterization of the Lithuanian cultivar ‘Gelsvis’ has been published; and available data is limited to morphological observations and distribution records (Bobinas, 2013; Drobná, 2010; Petrulaitis, 2022). Addressing these gaps is essential for sustainable agriculture, grassland restoration, and conservation of biodiversity in the Baltic Hemi-boreal region.

## 2 Methodology: literature search and synthesis strategy

This review was carried out methodically to compile the existing information related to the genetic diversity, the population structure, the distribution, and the agronomic of BFT in the Hemi-boreal region of the Baltic States. A detailed literature search was conducted in several scientific databases, Web of Science (Core Collection, Clarivate Analytics), Scopus (Elsevier), PubMed (National Library of Medicine), Google Scholar, CAB Abstracts (Centre for Agriculture and Biosciences International), and Science Direct (Elsevier). The choice of these databases aimed to offer a wide scope of peer-reviewed literature in plant genetics, molecular biology, ecology, and agronomy. The search plan has used Boolean operators to integrate the terms that are relevant to the species, genetic elements, and geographical setting. The main search terms were *L. corniculatus* (in a combination with genetic diversity or population structure) and *L. corniculatus* (in a combination with molecular markers or genetic variation), Birds-foot trefoil (in a combination with genetic diversity or germplasm), and (ISSR, SSR, AFLP or RAPD or SNP). The secondary search words were geographical and ecological: [*Lotus corniculatus* AND (Baltic States, Lithuania, Latvia, Estonia)], [*Lotus corniculatus* AND (Hemi-boreal or temperate or northern Europe)], [*Lotus corniculatus* AND (habitat or distribution or ecology or adaptation)], and [*Lotus corniculatus* and (breeding or conservation or forage legume)]. The individual database searches were tailored to each respective advanced search syntax of the databases and reference lists of retrieved papers were screened manually to come up with additional relevant literature not detected during the preliminary database searches.

The literature research included the period between January 2005 and November 2025, to ensure that the literature is as up to date as possible and that the research on modern molecular genetics is thoroughly covered, as well as the recent developments of genomic technologies and conservation genetics to be reflected. This deadline was chosen to limit the search to the modern literature using molecular markers and high-throughput sequencing methods, the last search is going to be conducted in November 2025. The literature was selected on the basis that it needed to be a study on BFT, or a closely related species within the *Lotus* genus, should contain empirical data on the genetic diversity, population structure, molecular characterization or ecotypic variation, cover ecological factors, habitat preferences, distribution patterns, or adaptive characteristics, and be in English. The exclusion criteria did not have enough details on methodology or reproducibility, concentrated on agronomic or physiological characteristics only but not genetic or molecular, non-peer-reviewed articles (conference abstracts, editorials, dissertations), duplicated datasets in other publications, or lack of relevance to the species BFT.

All the records retrieved were first filtered based on titles and abstracts to determine their relevance to the objectives of the review. Common records in databases were found and eliminated and the publications that could pass the initial screening criteria were evaluated on full text to ascertain their appropriateness based on the inclusion criteria. The last category of articles was used to establish qualitative synthesis in this review. The studies included were systematically extracted with the help of a systematic approach, and the following information was included: author(s), year of publication, journal; geographic origin of the population and sample size; type of molecular marker applied (e.g., AFLP, SSR, ISSR, RAPD, SNP etc.); genetic diversity parameters reported (e.g., heterozygosity [He, Ho], polymorphic information content [PIC], population differentiation [Fst]); ecological and environmental variables; habitat features and distribution data; agronomic traits and breeding implications; and the data that was extracted were sorted in summary tables to enable comparison across studies and geographical areas.

Since the literature reviewed has been heterogeneous in terms of both study designs and the use of different systems of markers as well as reported parameters, a qualitative synthesis method was chosen. The synthesis consisted of finding out the trends in research, genetic diversity and population differentiation patterns, common themes and gaps in knowledge. They had systematically compiled information on thematic topics such as distribution and habitat diversity, ecotypic variation, factors affecting genetic diversity, the use of molecular markers, population structure, conservation strategies, and agronomic implication. Results of the comparison and synthesis were made and used to obtain an in-depth knowledge about the BFT diversity, with specific reference to the Hemi-boreal zone and populations in Baltic States. The methodology of each included article was rated regarding the adequacy of the sample size, the validity of the molecular methodology used, the clarity of the genetic diversity presentation, and the reproducibility of the methods. Such a systematic methodology improves the level of transparency, reproducibility, and reliability of the review since it records the sources, search strategy, and criteria employed to gather and synthesize evidence on the genetic diversity of BFT clearly.

## 3 Distribution and habitat preferences in the Hemi-Boreal Zone

The distribution of BFT within the Hemi-boreal zone of the Baltic States is extensive, encompassing Lithuania, Latvia, and Estonia. This perennial legume demonstrates a broad ecological amplitude, thriving across diverse habitats including grasslands, meadows, roadsides, and coastal environments, reflecting its adaptability to varying edaphic and climatic conditions.

### 3.1 Large-scale distribution patterns in Europe

As a perennial minor leguminous crop, BFT occurs widely across Europe, Asia, and parts of Africa, and is distributed globally (Uysal, 2023). One of the significant questions is why BFT? Besides it can survive in the wild as well as local farm conditions (Chen et al., 2023). Owing to these agronomic and ecological attributes, BFT is widely cultivated for fodder production, soil restoration, and pasture improvement in agricultural fields and seminatural grasslands. The number of leguminous species increases towards southern floristic regions. The number of annual species also increases towards the south but remains lower than that of perennial species. No native annual legume species occur in the Arctic and Boreal zones, and even in the temperate Hemi-boreal and Atlantic regions only alien annuals are present (Szentesi, 2024). Within this context, BFT represents a widespread perennial species well adapted to northern and transitional European regions. In Latvia, BFT is recorded as a native species with high ecological adaptability. It grows across a wide range of soil conditions, from dry and nutrient poor soils to wet and fertile environments, enabling its establishment in meadows, roadsides, and other open or disturbed habitats. The species exhibits considerable morphological variation, which contributes to its ability to adapt to diverse environmental conditions and supports its role in local biodiversity (Drobná, 2010). Similarly, in Estonia, BFT shows broad distribution patterns, particularly in coastal and inland regions. In addition, European coastal and inland areas of Estonia contain *L. maritimus*, an alien species in the Baltic States, which survives across a wide soil range, including nutrient-poor, saline, and waterlogged environments (Petrulaitis, 2022).

### 3.2 Micro-habitat preferences and adaptations within the Hemi-boreal

In the Baltic States, BFT frequently occurs in regions where other legumes fail to maintain diversity under region-specific climatic conditions (King, 2023). This wide ecological distribution across diverse and often marginal environments suggests substantial adaptive genetic variation among populations, shaped by differential selection pressures related to soil chemistry, water availability, and climate. A widely distributed perennial legume, BFT occurs extensively across Lithuania, inhabiting inland, coastal, and northern regions of the country. The species can grow under both biotic and abiotic stresses, with its distribution influenced by edaphic and climatic factors (Petrulaitis, 2022; Sujkowska-Rybkowska et al., 2020). In the Hemi-boreal zone, BFT is found primarily in open habitats where full sunlight is available, which is crucial for its growth (Szentesi, 2024). In grasslands of the Hemi-boreal zone, BFT is usually associated with fertile sites. In open grasslands dominated by grasses, its occurrence is positively correlated with grazing activity and elevated soil sodium levels. Grazing intensity influences legume populations, including BFT, with growth often inhibited under excessively high grazing pressure (Abraham et al., 2015). The species’ deep taproot system enables access to subsoil nutrients and improves soil structure, making it particularly valuable for marginal land reclamation and mixed sward systems (Morris et al., 2021). As a legume, BFT enhances soil fertility and provides food resources for pollinators. It also supports diverse invertebrate communities and plays an important role in plant–insect interactions related to pollination and seed dispersal (Gudyniene et al., 2021). Due to these attributes, BFT is considered a valuable species for conservation and habitat restoration efforts in the Hemi-boreal zone (Gudyniene et al., 2021).

### 3.2 Key ecological drivers of distribution (climate, soil, disturbance)

The distribution of BFT across the Hemi-boreal zone is shaped by climatic conditions, soil properties, and disturbance regimes. Its ability to persist under diverse edaphic conditions including sandy, nutrient-poor, saline, and waterlogged soils contributes to its broad habitat range (Petrulaitis, 2022). In Lithuania, seminatural grasslands supporting BFT have undergone substantial land-use change. Between 2012 and 2014, surveys mapped 77,189 ha of meadows and EU-protected grassland habitats, of which approximately 9% were altered within four years, largely due to conversion to cropland, mixed plantations, and cultivated forests (Juknelienė et al., 2021). Long-term declines in permanent pastures, natural grasslands, and wetlands have been reported alongside increases in short-term grasslands and arable land expansion (Povilaitis et al., 2014). Agricultural intensification and habitat fragmentation therefore represent major disturbance factors threatening the persistence and genetic integrity of BFT populations. These pressures may lead to erosion of genetic diversity, emphasizing the importance of investigating population structure and adaptive variation as a basis for conservation and sustainable utilization.

## 4 Ecotypic and geographic variation

The broad distribution of BFT across the Hemi-boreal zone, spanning diverse habitats from coastal regions to inland grasslands and from nutrient-poor to fertile soils, establishes the foundation for understanding its adaptive capacity. This geographic distribution across environmentally heterogeneous landscapes creates distinct selection pressures that drive population differentiation. The interaction between climatic variables, edaphic factors, and anthropogenic activities does not affect all populations uniformly; rather, it creates a mosaic of environmental conditions that favor locally adapted genotypes (Živatkauskienė et al., 2024). Consequently, the geographical distribution patterns and habitat preferences documented in this section provide the ecological context necessary for understanding the ecotypic variation and genetic differentiation patterns that have evolved within these species across the Baltic States.

### 4.1 Gradient in morphological and life history traits

Ecotypic variability and geographic variation in BFT are evident across the Hemi-boreal region, which is characterized by mixed forests with cold, continental climates and often nutrient- poor soils. Species in this region have adapted through strategies such as symbiotic associations with mycorrhizal fungi and nitrogen-fixing rhizobia, and its is among the best-adapted legume species, demonstrating ecological plasticity under these challenging environmental conditions (Petrokas and Manton, 2023). Recent global resequencing studies have identified distinct population structure in BFT, though these studies lack representation from Baltic populations (Chen et al., 2023). The following groups do not include Baltic populations; their relevance to the Hemi-boreal zone of Baltic region requires validation through future molecular studies (Chen et al., 2023). Hemi-boreal zone consists of distinct groups. Group I is mainly distributed in Eastern Europe, such as Russia, Georgia, Ukraine, and Azerbaijan; Group II is mainly from Central Asia and West Asia, such as Georgia, Azerbaijan, and Kazakhstan; while Group III and Group Mix are mainly distributed in European countries and other continents (Manton et al., 2025). Subpopulations exhibit significant differences in geographical distribution and agronomic characteristics. Group I has the lowest plant height and stem length but the highest cyanogenic glycoside content; Group II has the highest plant height and stem length but the lowest cyanogenic glycoside content; Group III has intermediate biomass and the lowest cyanogenic glycoside content (Chen et al., 2023). Similarly, BFT plants from different regions show variation in morphological characteristics, growth habits, indumentum, leaf shape, and reproductive capacity (Merkouropoulos et al., 2017). Plants belonging to different ecotypes also exhibit variation in growth, productivity, and yield components under different soil moisture conditions (Merkouropoulos et al., 2017). Altitude-related variation is also evident. Lower-altitude populations tend to produce semi- prostrate plants with longer stems and later flowering times. As population collection sites shift westward and southward, plants display more stems, more internodes, and longer stems. Geographical isolation contributes to genetic differentiation among populations, which is a key factor underlying geographical differences in morphological traits and ecological adaptability (Abraham et al., 2015). At higher altitudes, populations may experience stronger ultraviolet radiation and lower temperatures, leading to selection for genes associated with stress resistance (Abraham et al., 2015). The observed ecotypic variability including differences in plant height, stem length, flowering time, cyanogenic glycoside content, and stress resistance reflects underlying genetic differentiation among populations. These phenotypic differences represent adaptive responses to local environmental conditions shaped by natural selection acting on genetic variation (Abraham et al., 2015).

### 4.2 Environmental gradients driving phenotypic plasticity vs. genetic differentiation

In the Hemi-boreal zone, ecological factors strongly modulate the distribution of BFT. Climatic variables, particularly temperature and precipitation, play a central role by directly affecting soil moisture dynamics. Differences in soil moisture regulate the mineralization and mobility of soil organic carbon and consequently influence nutrient availability and habitat suitability (Petrulaitis, 2022). As a highly adaptable plant, BFT can maintain relatively high yields under poor, saline, and flooded conditions, indicating considerable adaptability to climatic variation (Chen et al., 2023). Climate change effects on vegetation phenology depend on geographical location, topography, and soil type, and further studies are required to understand how these factors interact (Guo et al., 2024). Soil structure provides space, air, nutrients, and water for plant growth, and soil pore characteristics regulate temperature, airflow, and water availability, thereby indirectly influencing vegetation growth (Guo et al., 2024).

The spread of BFT is closely linked to human activities, including soil improvement through nitrogen incorporation and the distribution of different germplasms driven by human selection (Chen et al., 2023). Species composition and genetic diversity of BFT are influenced by management practices such as grazing, with higher diversity observed in areas with minimal grazing pressure, particularly forest edges (Abraham et al., 2015). Agricultural practices in Lithuania, including land-use changes and environmental modification, have reduced the size and diversity of permanent grasslands, with consequences for forage productivity and quality. In the Hemi-boreal region, climatic factors are therefore critical when forecasting changes in the distribution and abundance of BFT (Živatkauskienė et al., 2024). In addition, interactions with invasive species, such as Impatiens parviflora, highlight the importance of understanding species–environment relationships in the Hemi-boreal Baltic zone ( (Meištininkas and Žaltauskaitė, 2024).

Environmental and climatic factors such as temperature, precipitation, and soil conditions directly influence growth, stress tolerance, and gene expression in BFT. Temperature affects growth rates, carbohydrate reserves, and phenolic profiles, influencing regrowth potential and adaptation. While moderate temperature changes alone may not affect forage quality, interactions with elevated CO_2_ or drought can increase phenotypic and genetic variation (Morris et al., 2021). Precipitation regimes also shape genetic diversity, with stable moisture conditions supporting richer and more stable gene pools, whereas water limitation imposes strong selection pressures (Abraham et al., 2015). Local genotypes have developed physiological and genetic traits that enhance tolerance to water stress. Genotypes capable of maintaining higher relative water content and stable chlorophyll levels under drought conditions demonstrate improved adaptability to water-limited environments (Bostan et al., 2025). Soil nitrogen availability further influences growth and biomass production, indirectly affecting the expression of genetic variation in legumes capable of symbiotic nitrogen fixation (Gonnami et al., 2024). Together, these environmental gradients interact with genetic differentiation to produce the phenotypic plasticity observed across BFT populations, shaping both short-term adaptive responses and long-term evolutionary trajectories.

## 5 Genetic diversity: patterns and drivers

Genetic diversity is a fundamental driver of plant adaptation and evolutionary processes. It represents the total amount of genotypic and phenotypic variation within and among populations and reflects the balance between mutation and loss of genetic variation (Amiteye, 2021). Genetic diversity underpins evolutionary potential, environmental responsiveness, and long-term species persistence. Molecular markers are widely employed to detect DNA polymorphisms and accurately differentiate between individuals within and between populations. Despite their utility, comprehensive analyses of Lotus corniculatus populations in the Baltic States remain limited.

### 5.1 Choice and application of molecular markers for assessment of genetic variation in plant species

**Figure 1:**
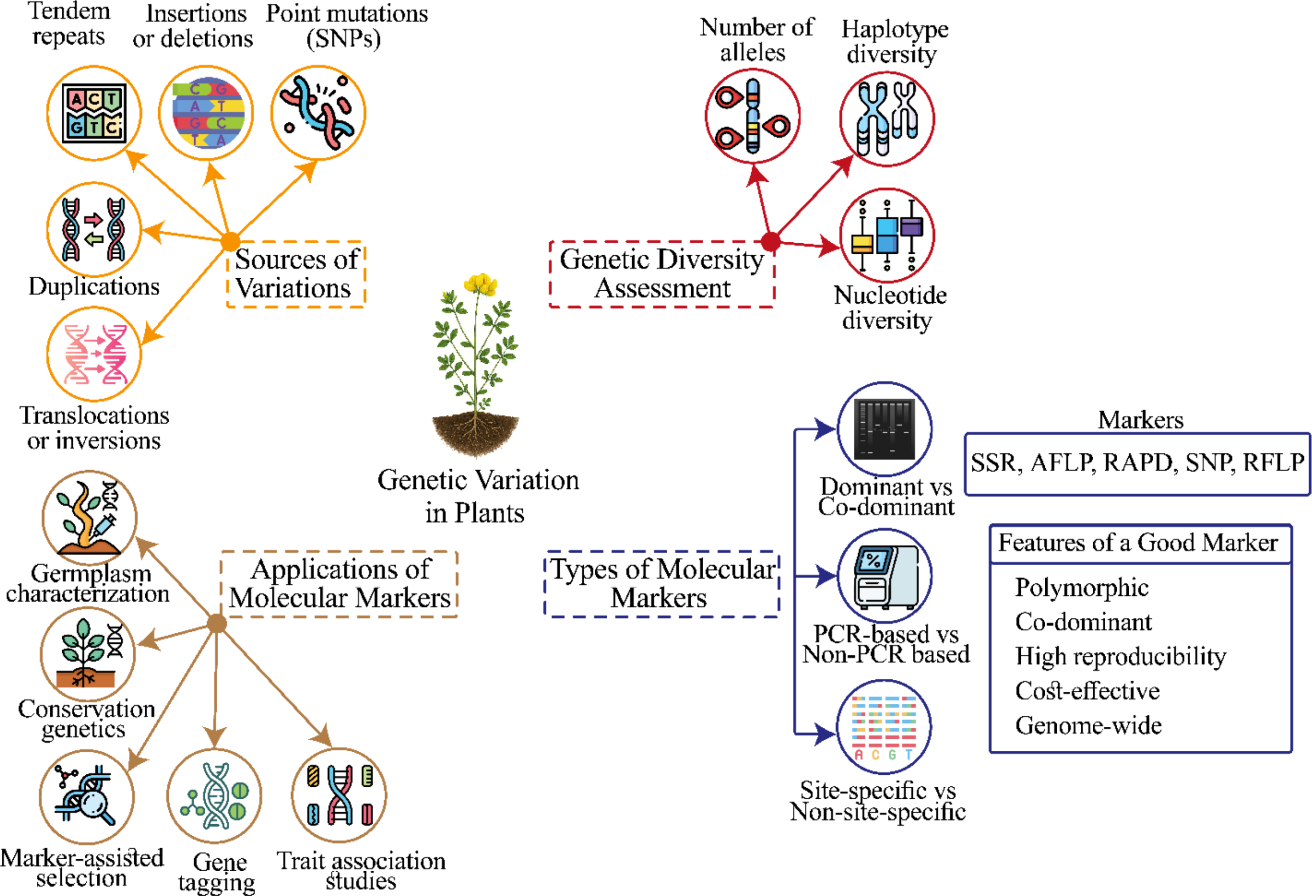
Impact of anthropogenic activities on genetic diversity and population adaptability in plants.

The use of genetic diversity in plant populations is based on the molecular markers to identify the polymorphisms of DNA and distinguish between individuals within and between populations. Examples of molecular markers are PCR-based, non-PCR-based systems, dominant or co-dominant patterns of inheritance, site-specific or genome-wide use (Amiteye, 2021). Although there are various platforms of markers (**Table 1**), their use has been unevenly distributed over geographical areas with significant gaps in Baltic Hemi-boreal populations. Various molecular marker systems have successfully characterized BFT genetic diversity globally: Mediterranean populations studied using AFLP, SSR, and RAPD markers (Giagourta et al., 2015; Merkouropoulos et al., 2017); Eastern European (non-Baltic) populations characterized with ISSR markers documenting hybridization (Kramina, 2013; Kramina et al., 2018); and worldwide resequencing of 324 accessions identifying population structure and genetic diversity centers in Transcaucasia (Chen et al., 2023). However, no Baltic accessions were included in these comprehensive studies. A molecular or DNA marker is the difference in DNA nucleotide sequence between individual organisms or species that are in proximity or tightly linked to a target gene to express a trait. Typically, the target gene, expressed trait or biological function, and the associated tightly linked molecular marker are inherited together (Amiteye, 2021). DNA markers are useful for identifying genotypic differences within or between species when variations, known as polymorphisms, occur in the nucleotide sequences of the markers. Molecular marker polymorphisms are due to varied types of DNA mutations that create nucleotide sequence differences between or among organisms (Amiteye, 2021). Generally, marker polymorphisms in organisms are caused by point mutations arising from single nucleotide substitutions, rearrangements involving insertions or deletions, DNA section duplication, translocations, and inversions, as well as mistakes in replication of DNA that are tandemly repeated (Amiteye, 2021). Molecular marker signals that are used to reveal genotypic differences between individuals due to marker sequence differences are called polymorphic markers. On the other hand, DNA markers that cannot be used to differentiate between or among genotypes are referred to as monomorphic markers. The characteristics of a good and very useful DNA marker are that the marker is ubiquitous and evenly distributed throughout the genome, easy to assay, cost-effective, multiplexed, and can be automated. An ideal molecular marker must also be highly polymorphic and co-dominant in expression to enable effective discrimination between homozygotes and heterozygotes and should be highly reproducible and possible to share data generated among laboratories. Additional characteristics of a very good molecular DNA marker are that the marker creates no detrimental effect on phenotype, is genome-specific in nature, and is multi-functional (Amiteye, 2021). (**Table 1**, **Figure 1**).

**Table 1:** Molecular marker type, advantages, disadvantages, mechanisms of action and their applications in minor legumes and *L. corniculatus*.

Marker	Type	Mechanism of action	Application in minor legumes & *Lotus corniculatus*
AFLP (Amplified Fragment Length Polymorphism)	DNA Restriction based (Jajoriya et al., 2025)	DNA digestion, ligation & selective, amplification (Jajoriya et al., 2025)	Mapping of agronomic traits and analyze the genetic variation of *L. corniculatus* Intra- and inter-population diversity (Kramina, 2013) highlights the effectiveness in distinguishing closely related basil genotypes and demonstrate the rich genetic resources present in the Croatian basil germplasm (Varga et al., 2024)
RFLP (Restriction Fragment Length Polymorphism)	DNA restriction based (Jajoriya et al., 2025)	DNA digestion, ligation & selective, amplification (Abraham et al., 2015)	Created first genetic linkage map in *L. corniculatus*. Assessing genetic diversity in *Mucuna pruriens.* (Doveri et al., 2024)
CAPS (Cleaved Amplified Polymorphic Sequences)	DNA restriction based (Ali et al., 2024).	DNA digestion, ligation & selective, amplification (Brant et al., 2024)	Breeding programs for minor legumes improvement against biotic and abiotic stresses (Maurya et al., 2025)
RAPD (Random Amplified Polymorphic DNA)	Based on random amplification (Li et al., 2021)	DNA digestion, ligation & selective, amplification (Amiteye, 2021)	Genetic diversity in minor legumes and Identify genetic variation and breeding in *L. corniculatus* (Abraham et al., 2015; Babu et al., 2020)
Inter-Simple Sequence Repeats (ISSR)	Based on random amplification. (Gemmill and Grierson, 2020)	Anchored PCR amplification (Gemmill and Grierson, 2020)	Study genetic structure & diversity in mungbean. Genetic diversity *in L. corniculatus*. (Gemmill and Grierson, 2020; ShipSiddique and Jeelani, 2015)
RAMP (Random Amplified Microsatellite Polymorphism) Marker	PCR-based DNA (Liu et al., 2021)	Selective amplification of IRAP–SSR regions (Liu et al., 2021)	Identify Genetic diversity Identify QTLs traits forage quality and disease resistance. Supports germplasm conservation (Rani et al., 2024)
DALP (Direct amplification of length polymorphism)	DNA-based molecular (Amiteye, 2021)	Selective amplification of polymorphic loci (Amiteye, 2021)	Development of linkage maps. Aiding marker-assisted breeding in stress-resistant varieties. (Chandra et al., 2024)
SCAR (Sequence Characterized Amplified Region)	PCR-based (Amiteye, 2021)	PCR amplification of specific loci (Amiteye, 2021)	Gene mapping and marker-assisted selection. Improvement of disease resistance in Vicia and Lens genera. (Chandra et al., 2024)
Simple Sequence Repeats (SSRs)	PCR-based (Kumar et al., 2022)	PCR amplification of microsatellites (Mishra et al., 2022)	Genetic diversity assessment, gene mapping, marker-assisted selection, and disease control in minor legumes. Variability among cultivars in *L. corniculatus* (Chaudhary et al., 2019)
Chloroplast Simple Sequence Repeats (cpSSRs)	Based on microsatellite repeats located cpDNA. (Guo et al., 2022)	PCR amplification of cpSSR loci (Yin et al., 2022)	In minor legumes like *Lotus* spp.it helps in preserving genetic diversity, improving drought resistance, and nutrient efficiency. In *L. corniculatus* it reveals genetic variation for adaptability. (Chen et al., 2023)
Sequence-related amplified polymorphism (SRAP)	PCR-based (Jajoriya et al., 2025)	Open reading frame–targeted amplification (Jajoriya et al., 2025)	Determining the genetic variability of lentils. To examine the genetic variation and population structure of different legume species. (Mohammed et al., 2023)
Sequence-Tagged Site (STS)	PCR-based (Bhatia and Bajwa, 2022)	DNA digestion, ligation & selective amplification (Jajoriya et al., 2025)	In *L. corniculatus*, it is used for genetic diversity studies. GWAS analysis for traits like cyanogenic glycoside content, and population structure analysis. (Chen et al., 2023)
SCoT (Start Codon Targeted) markers	PCR-based (Ivanovska et al., 2025)	DNA digestion, ligation & selective amplification (Ivanovska et al., 2025)	No specific information related to minor legumes and *L. corniculatus.* Used in various plant species for genetic diversity analysis and characterization (Ivanovska et al., 2025)
SNPs (Single Nucleotide Polymorphisms)	Non-restriction-based (Amiteye, 2021)	Single base variation detection (Amiteye, 2021).	Used in minor legumes for trait mapping (e.g., drought resistance, disease resistance). In *L. corniculatus*, it aids in determining stress tolerance and crop improvement. (Chen et al., 2023)
SSCP (Single-Strand Conformation Polymorphism)	PCR-based (Singh, 2024)	DNA denaturation & conformations (Kakavas, 2021)	Detecting genetic variation in alfalfa genes and in *Vicia Faba* L., genetic linkages, genetic diversity of rare and endemic species. (Al-Yasiri et al., 2021)
IRAP (Inter-Retrotransposon Amplified Polymorphism)	Retrotransposon-based molecular marker (Ramakrishnan et al., 2023)	Primer binding & amplification (Kalendar, 2015)	Conducting genetic diversity studies in minor legumes. Assessing genetic diversity in *L. corniculatus*. (Kramina et al., 2018; Tomás et al., 2016)
REMAP (Retrotransposon Microsatellite Amplification Polymorphisms)	DNA-based marker. (Amiteye, 2021)	Retrotransposon primers amplification (Prasad et al., 2022)	In minor legumes and in *L. corniculatus* includes uncovering genetic diversity, aiding breeding programs. (Aşkar and Bilgen, 2023; Karadağ and Seydoşoğlu, 2023)
Retrotransposon-based insertion polymorphism (RBIP)	DNA-based molecular (Arvas et al., 2021)	Transposon insertion detection (Ramakrishnan et al., 2023)	Aiding breeding programs, variety identification, and germplasm management in *L. corniculatus.* (Meng et al., 2021)
iPBS (inter-Primer Binding Site)	Retrotransposon-based (Bidyananda et al., 2024)	PCR amplification using retrotransposon primers (Arvas et al., 2021)	Genetic studies are highly applicable in *L. corniculatus and* other species like Daucus, flax, and Fagaceae (Bidyananda et al., 2024; Kizilgeci et al., 2022)
Target Region Amplification Polymorphism (TRAP)	PCR-based marker (Khidr et al., 2020)	PCR of target regions (Khidr et al., 2020)	Finding genetic diversity in minor legumes. In *L. corniculatus*, it can be used for genotyping and tagging candidate genes controlling traits. (Khidr et al., 2020)
Diversity Array Technology (DArT) markers	Dominant and high throughput (Ma and Grima, 2025)	Complexity reduction & hybridization (Deresa and Diriba, 2023)	Accelerating breeding programs and enhancing genetic diversity studies, including minor legumes and *L. corniculatus.* (Zhao et al., 2024)
EST-SSRs (Expressed Sequence Tag-Simple Sequence Repeats)	Gene-based (Sharma and Singh, 2021)	PCR amplification of expressed sequences (Sharma and Singh, 2021)	Assessing genetic variation, identifying trait-linked markers, and supporting breeding programs in *L. corniculatus* and other minor legumes. (Chen et al., 2015)

### 5.2 Spatiotemporal patterns of genetic diversity in the Baltic region

The genetic diversity of minor legumes, including BFT, in the Baltic region exhibits substantial spatial variation influenced by geographic and ecological factors. This widely distributed perennial legume demonstrates high levels of genetic diversity, which contributes to its adaptability and ecological restoration potential. Genetic diversity is often structured into distinct subgroups, with major diversity centers reported in regions such as Transcaucasia (Chen et al., 2023). In the Baltic region, studies on *Trifolium fragiferum* in Latvia revealed lower genetic diversity in wild populations compared to cultivated varieties, along with distinct genetic clusters among populations (Ruņģis et al., 2023). Similarly, BFT populations exhibit considerable genetic variation both within and among populations, which supports adaptation to diverse environments (Chen et al., 2023). Genetic diversity in BFT is also influenced by geographic distance and ecological characteristics. Genotypes adapted to similar environments may display phenotypic similarity despite geographic separation (Merkouropoulos et al., 2017). In regions such as Russia and Ukraine, hybridization between BFT and related species further contributes to genetic diversity, with evidence of gene flow and transitional morphologies (Kramina, 2013).

### 5.3 Landscape genetic evidence for environmental-climatic drivers

Environmental heterogeneity, geographic distance, and ecological gradients play a significant role in shaping genetic diversity and population structure. Studies using molecular markers such as AFLP, SSR, and ISSR have provided insights into genetic relationships, differentiation, and population structure across landscapes (Nazir et al., 2021). These approaches reveal how environmental and climatic factors influence gene flow, local adaptation, and genetic clustering in plant populations, including BFT.

### 5.4 Anthropogenic effects (land-use change, management practices)

**Figure 2:**
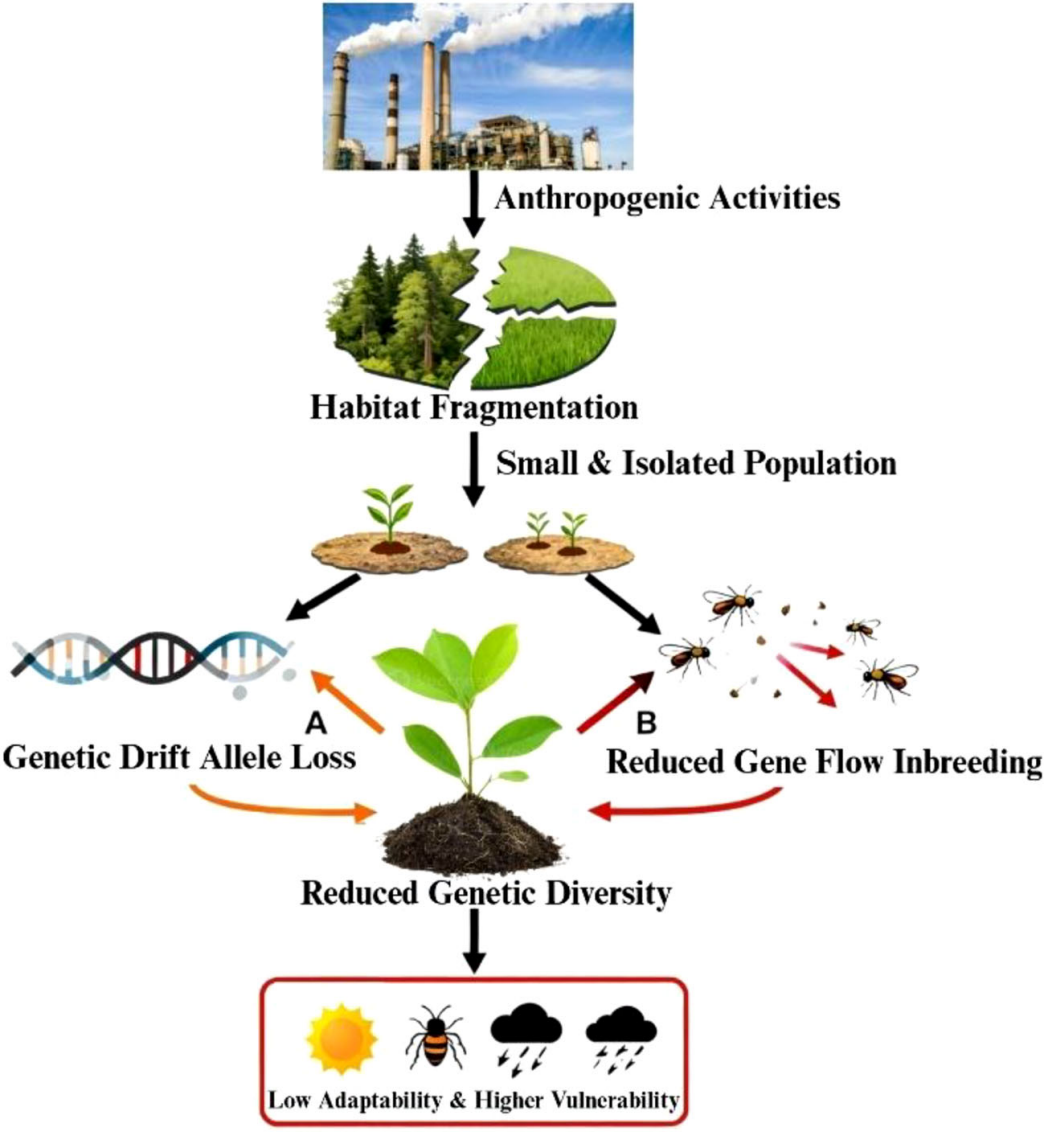
Biotic factors such as species interactions, soil microbes, trophic dynamics, and microevolutionary processes shape plant genetic diversity, structure, and resilience. These interactions influence gene expression, adaptation, and ecological stability across multiple trophic levels.

Anthropogenic factors such as monoculture farming, habitat loss, land-use change, and underutilization of germplasm pose serious threats to plant genetic diversity. These pressures reduce genetic variation, increasing vulnerability to biotic and abiotic stresses (**Figure 2**) (Gepts, 2023). In agricultural systems, limited use of diverse genetic resources constrains breeding potential and resilience. Conservation of genetic resources and sustainable management practices are therefore essential for maintaining genetic diversity in BFT and other minor legumes.

### 5.5 Biotic factors (pollinators, herbivores, symbiotic microbes)

**Figure 3:**
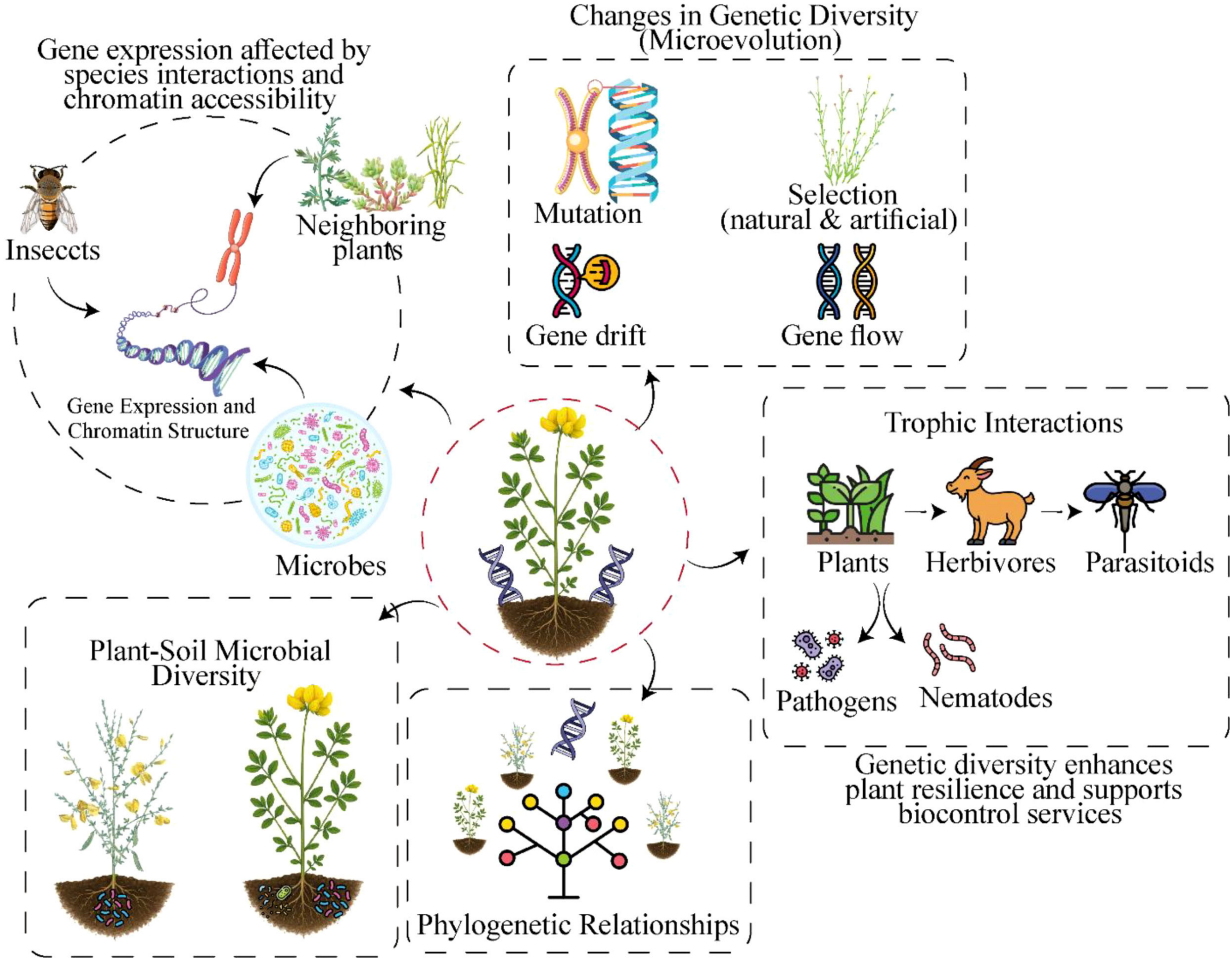
Molecular marker types, applications, features and their assessment.

Biotic interactions strongly influence genetic diversity and population structure in plant species. Pollination mechanisms, seed dispersal strategies, herbivory, and interactions with symbiotic microbes affect gene flow, reproductive success, and genetic differentiation. Habitat fragmentation alters these interactions, leading to changes in population connectivity and genetic structure. The combined effects of pollinators, dispersal agents, and microbial symbioses contribute to the evolutionary dynamics and ecological adaptability of BFT and related forage legumes (**Figure 3**).

### 5.6 Genetic diversity studies in BFT in the Baltic States

Several studies have assessed genetic diversity in crops using molecular markers across Europe and the Baltic region. Genetic diversity in natural BFT populations has been investigated using morphological traits and molecular markers such as AFLP, SSR, and RAPD (Giagourta et al., 2015). As a minor but valuable forage legume, understanding its genetic diversity is critical for breeding and conservation. **Table 2** summarizes genetic diversity patterns observed across minor legumes and forage crops, providing context for BFT research.

**Table 2:** Application of different DNA markers in minor legumes and grasses.

Plant	Molecular marker application	Type of molecular marker	References
Birdsfoot trefoil (*Lotus corniculatus* L.)	Genetic diversity	ISSRs	(Abraham et al., 2015)
Alfalfa (*Medicago sativa* L.)	Genetic diversity, genetic structure	ISSR: SSRs	(Heiba et al., 2022)
Red Clover (*Trifolium pratense* L.)	Gene flow and Genetic diversity	ISSR	(Petrauskas et al., 2023)
white clover (*Trifolium repens* L.)	Genetic diversity	SCAR; RAPD	(Han et al., 2022)
Mungbean (*Vigna radiata*)	Genetic diversity & QTL mapping,	SSRs (EST-SSRs), SNPs, GBS	(Wu et al., 2017)
Bambara groundnut (*Vigna subterranea*)	Genetic diversity & Population structure,	DArTseq, SSRs, SNPs	(Uba et al., 2021)
Pigeon pea (*Cajanus cajan*)	Genetic diversity & SNP discovery	SNPs (GBS), SSRs	(Kinhoégbè et al., 2022)
Timoty (*Phleum pratense* L.)	Genetic diversity	SSR	(Tanaka et al., 2015)
Smooth bromegrass (*Bromus inermis*)	Genetic diversity & GWAS/trait-association	SRAP, SNPs, GWAS/GBS approaches	(Saeidnia et al., 2022)
Bromegrass (*Bromus inermis*)	Genetic diversity & Genetic variation	AFLP and RAPD	(Antonova and Roeder, 2022)
Napier grass (*Pennisetum purpureum*)	Genetic diversity & Germplasm characterization	GBS/SNPs, SSRs	(Muktar et al., 2019)
Kikuyu grass (*Pennisetum clandestinum*)	Genetic diversity & cultivar identification	SNPs (DArT/GBS) and SSR	(Pudzianowska et al., 2020)
Orchardgrass (*Dactylis glomerata* L.)	High polymorphism & genetic variability	SSR	(Nabhan et al., 2023)
canary grass (*Phalaris canariensis* L.)	Genetic diversity & molecular-assisted breeding	EST-SSR	(Jia et al., 2023)
Meadow fescue (*Festuca pratensis*), Annual ryegrass (*Lolium multiflorum*), Red fescue (*Festuca rubra*), Festulolium (*Festulolium*)	Genetic diversity & Genetic polymorphism	SCoT	(Mavlyutov et al., 2021)
Meadow bromegrass (*Bromus biebersteinii*)	Genetic diversity and transcriptomic resources	SSR, AFLP, SNPs	(Sun et al., 2021)
Quackgrass (*Elymus repens*)	Genetic diversity & variety identification	AFLP, SSRs, chloroplast markers	(Fahleson et al., 2008)
Cocksfoot/Orchardgrass (*Dactylis glomerata*)	Genetic diversity & trait association	SSRs, SRAP, SNPs (GBS)	(Hamstra, 2016)
Perennial ryegras (*Lolium perenne*)	Genetic diversity & Polymorphism	DArT, SNP, and SSR,	(Liu, 2016)
Blue grama (*Bouteloua gracilis*)	Population structure, restoration genetics	AFLP, RAPD, cpDNA, SNPs/GBS	(Tso and Allan, 2019)
Vetches (*Vicia* spp.)	Genetic diversity, breeding, phylogenetics	SSRs, ISSRs, SNPs	(Bidyananda et al., 2024)
Bentgrass (*Agrostis capillaris*)	Genetic diversity and interspecific hybridization	SSRs	(Li et al., 2023)
Kentucky bluegrass Or Meadow grass (*Poa pratensis*)	Genetic diversity	RAPD and ISSR	(Szenejko et al., 2016)
Fine Fescue (*Festuca ovina* L.)	Genetic diversity	ISSR	(Mohammadi et al., 2020)
Prairie cordgrass (*Spartina pectinata*)	Germplasm characterization, SNP validation	SNPs (KASP), SSRs, cpDNA markers	(Graves et al., 2016)
hard fescue (*Festuca brevipila*)	Genetic diversity	SNP	(Bushman et al., 2024)
Creeping bentgrasses (*Agrostis stolonifera*)	Genetic Differentiation	SSR	(Zapiola and Mallory-Smith, 2017)
Bur medic (*Medicago minima*)	Genetic diversity	SSR	(Bagheri et al., 2022)
Faba bean (*Vicia faba*)	Genetic diversity & trait mapping	SNP arrays, GBS, SSRs	(Skovbjerg et al., 2023)
Lupins (*Lupinus* spp.)	Genetic diversity	SNPs (GBS), SSRs	(Tosoroni et al., 2025)
Sesbania (*Sesbania* spp.)	Genetic diversity	SNPs, SSR, GBS	(Negawo et al., 2022)
Desmanthus (*Desmanthus* spp.)	Genetic diversity & cultivar identification	ISSR, SSRs, SNPs	(Costa et al., 2017)
Winged bean (*Psophocarpus tetragonolobus*)	Genetic diversity & linkage map	SSR, ISSR, SNPs; first linkage map & QTLs	(Chankaew et al., 2022)
Horse gram/Macrotyloma (*Macrotyloma uniflorum*)	Genetic diversity	RAPD, ISSR, SSR, SNPs	(Jha et al., 2022)
Aeschynomene (*Aeschynomene* spp.)	Phylogenetics & Genetic diversity	SSR, AFLP, SNPs	(Bidyananda et al., 2024)

Despite the potential applicability of various molecular tools, five major limitations hinder their effective use in the region. First, standardized SSR panels specific to Baltic germplasm are absent, restricting regional comparisons despite existing northern Eurasian datasets (Kramina, 2013). Second, no SNP-based population genomic analyses have been conducted, although such approaches are widely applied elsewhere (Chen et al., 2023). Third, adaptive traits lack associated functional markers, as no QTL or association studies have been undertaken using Baltic ecotypes. Fourth, the phylogeographic resolution of existing data is insufficient; chloroplast sequencing could clarify whether Baltic populations represent unique glacial refugia or postglacial expansions, aiding conservation prioritization. Fifth, molecular and phenotypic datasets remain unintegrated, limiting the ability to link genotypic variation with field performance traits such as winter survival, regeneration, and stress tolerance.

These methodological gaps constrain the efficiency of breeding programs. The successful introduction of Lithuanian cultivar Gelsvis in the 1950s via the use of phenotypic selection (Bobinas, 2013), proves that the latter can be enhanced; nonetheless, the breeding process can be improved exceptionally fast by using modern marker-assisted selection (MAS). MAS permits early choice in winter hardiness, inbreeding tracking, and recognition of heterotic groups. To achieve these benefits, the QTL mapping and GWAS should be put in place regarding the Baltic germplasm to determine markers traits associations.

To systematically address these limitations, a phased research strategy is proposed. Phase I (years 1–2) should focus on baseline diversity assessment using ISSR markers across 50–100 populations from Lithuania, Latvia, and Estonia. Although ISSRs offer lower resolution than SSRs or SNPs, they provide a cost-effective and rapid means to characterize genetic variation and identify priority populations for subsequent in-depth molecular analyses in later phases.

## 6 Population structure and gene flow

Population structure and gene flow are central to understanding the evolutionary dynamics, adaptability, and long-term persistence of plant species. In BFT and other minor legumes, the genetic makeup of populations is shaped by ecological, geographic, and evolutionary factors that determine environmental responsiveness. Processes such as pollination, seed dispersal, habitat fragmentation, and landscape heterogeneity influence population connectivity and genetic differentiation. Conversely, monoculture practices, habitat loss, and underutilization of germplasm reduce genetic variation, increasing vulnerability to biotic and abiotic stresses. Consequently, conservation of genetic diversity and germplasm preservation are essential for sustainable agriculture, plant breeding, and ecological resilience.

### 6.1 Geographic barriers and landscape connectivity

Geographic and landscape features play a critical role in structuring plant populations by regulating gene flow and genetic connectivity. The genetic diversity of minor legumes, including BFT, in the Baltic region is characterized by pronounced spatial variation driven by geographic distance, ecological heterogeneity, and physical barriers. As a widely distributed perennial legume, BFT exhibits high genetic diversity, which underpins its adaptability to diverse environments and its potential for ecological restoration. This diversity is often structured into distinct genetic subgroups, with major diversity centers reported in regions such as Transcaucasia (Chen et al., 2023). Within the Baltic region, landscape fragmentation and regional isolation contribute to differentiated population structures. For example, wild populations of *Trifolium fragiferum* in Latvia show lower genetic diversity compared to cultivated varieties, with clear genetic clustering among populations, reflecting restricted connectivity and localized adaptation (Ruņģis et al., 2023). Similarly, BFT populations demonstrate substantial genetic variation both within and among populations, highlighting the influence of geographic and ecological gradients on population differentiation (Chen et al., 2023).

Geographic distance and environmental dissimilarity further shape genetic patterns, as genotypes adapted to similar ecological conditions may exhibit phenotypic resemblance despite spatial separation (Merkouropoulos et al., 2017). In addition, natural hybridization between BFT and related species in regions such as Russia and Ukraine contributes to genetic diversity and landscape-level gene flow, producing transitional morphologies and evidence of introgression (Kramina et al., 2021). Molecular marker-based studies using AFLP, SSR, and ISSR have been instrumental in revealing these spatial patterns, providing insights into genetic relationships, differentiation, and population structure across fragmented landscapes (Nazir et al., 2021).

### 6.2 Relative contributions of pollen vs. seed dispersal to gene flow

Gene flow in BFT is mediated by both pollen and seed dispersal, with their relative contributions shaped by biological traits, landscape structure, and environmental constraints. Across Europe, including the Baltic States, BFT is recognized for its genetic diversity and adaptability; however, genetically distinct populations indicate limitations in effective gene exchange (Chen et al., 2023). Pollen-mediated gene flow is influenced by pollinator behavior and habitat configuration. Bumblebees, the primary pollinators of BFT, exhibit limited movement between forage patches, with less than approximately 2.6% inter-patch pollination, resulting in restricted pollen flow and increased within-population mating (Wenzell, 2021). This constrained pollen dispersal reduces genetic connectivity among populations, particularly in fragmented landscapes.

Seed dispersal also contributes to gene flow but is generally characterized by short dispersal distances, imposing further limitations on long-range genetic exchange (Chiu et al., 2023). In fragmented habitats, reduced seed dispersal effectiveness amplifies the effects of genetic drift, altering population genetic structure and increasing differentiation (Chiu et al., 2023). These findings suggest that isolation alone does not fully explain observed genetic patterns; rather, the interaction between limited dispersal mechanisms and landscape fragmentation plays a decisive role. Additional factors, such as geographic barriers including the Baltic Sea, rivers, urban development, and agricultural intensification, further restrict both pollen and seed movement across the region (Chiu et al., 2023). Moreover, shifts from cross-pollination to increased autogamy observed in some BFT populations reduce gene flow and exacerbate genetic differentiation, reinforcing population structuring at local (Kramina et al., 2021).

### 6.3 Threats to genetic diversity

**Figure 4:**
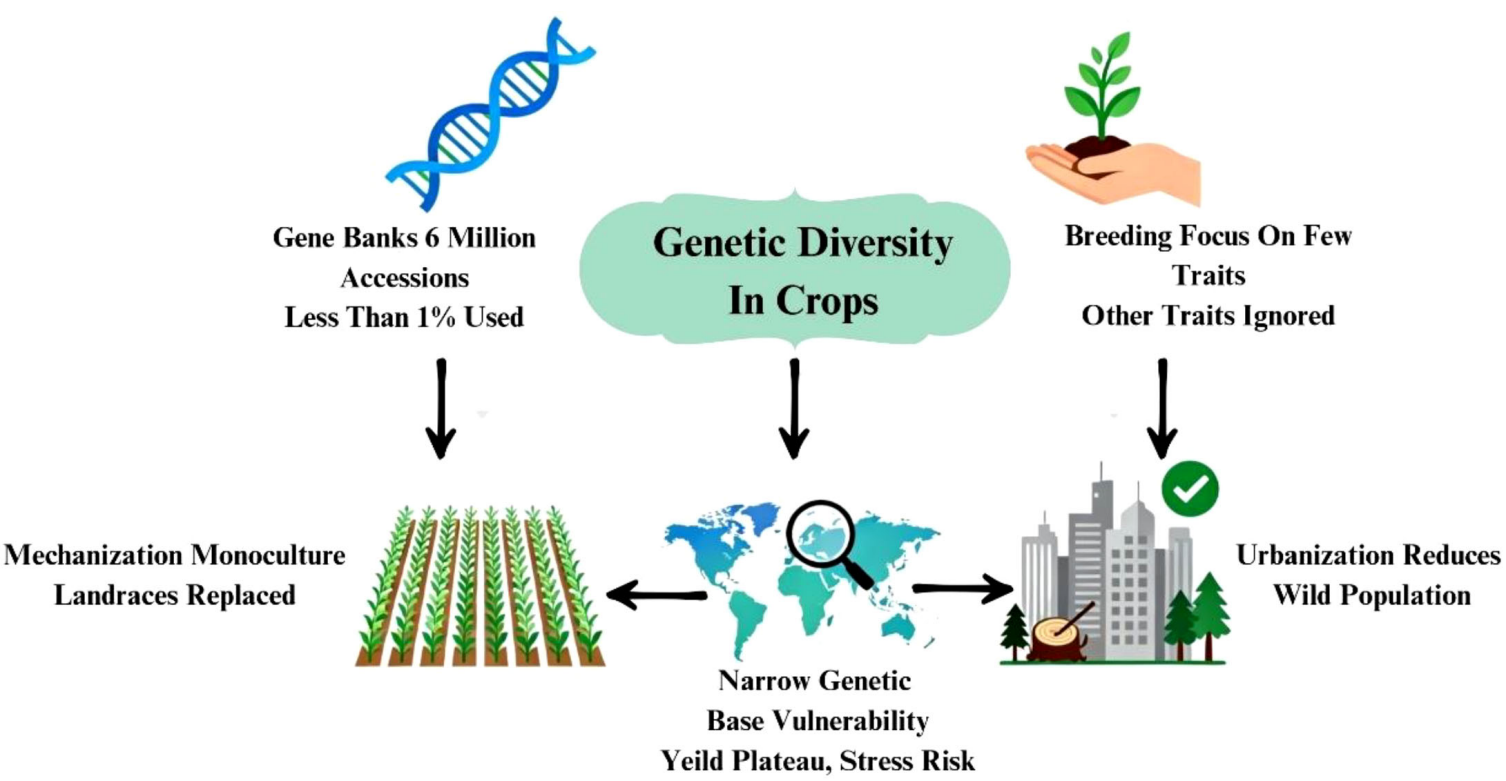
Factors contributing to the erosion of genetic diversity in crops.

Gene banks across the world maintain much germplasm (about 6million) of important crop plants; of them, less than 1% has been utilized by breeders (Bhandari et al., 2017). This is because of the lopsided approach of plant breeding, aiming at only a few important traits contributing towards yield at the cost of other traits (Lamichhane and Thapa, 2022). Many other germplasm accessions possessing diverse traits remain unutilized. This leads to a narrow genetic base of crop varieties, leading to genetic vulnerability which, may be devastating in the context of changing climatic conditions (Bhandari et al., 2017). Increased mechanization in agriculture has paved the way for monoculture over a large tract of land. This has replaced many landraces and local varieties from the farmers’ fields, which are the genetic reservoirs of many useful traits (Bhandari et al., 2017). Apart the destruction of natural habitats in the name of urbanization and modernization it has reduced the scope of generating natural variation in the form of wild forms and wild relatives of crop plants (Bhandari et al., 2017). With the commercialization in agriculture, a few lines have been used exhaustively in breeding new varieties/hybrids almost to the exclusion of others (Bhandari et al., 2017). This has resulted in yield plateauing and susceptibility of these varieties to different biotic and abiotic stresses. Genetic diversity in the form of different landraces and germplasm serves as the source of important genes for biotic and abiotic stresses (**Figure 4**) (Bhandari et al., 2017).

## 7 Ecology of BFT in the Hemi-boreal zone

The ecology of the Hemi-boreal zone is determined by the specific climatic, edaphic and vegetation features of the region that affect its distributions and adaptability. It is a widespread seminatural legume, which grows in various areas, such as meadows, grasslands, and wetlands, where average moisture levels and low nutrient content encourage its symbiotic fixation of nitrogen. As an ecological element, BFT plays a vital role in the operation of an ecosystem due to its role as a pollinator, as well as its ability to enrich the soil by fixation of nitrogen, and to support fauna associated with its presence, improving biodiversity and ecological stability.

### 7.1 Characteristics of the Hemi-boreal zone

In the Hemi-boreal zone, climatic characteristics, including temperature, precipitation, and humidity, are evident. The region experiences a cool to mild climate, with winter temperatures frequently dropping below freezing and summer temperatures ranging between 15 °C and 20 °C (Kisić et al., 2021). Climate is characterized by elevated relative humidity, typically above 70%, creating a moist environment that supports the growth and persistence of various plant species (Brazaitytė et al., 2025). Similarly, soil characteristics include soil composition, nutrient availability, and permafrost (Brazaitytė et al., 2025). One of the important characteristics of soil composition is pozole because it has average leaching and is very vulnerable to compaction and provides availability of organic compounds. Nutrient presence is scarce in this region because organic layers provide nutrients through the decomposition process (Brazaitytė et al., 2025). Permafrost is known as frozen soil, rock or sediment which is usually observed in Hemi-boreal zone (Wei et al., 2024). Thirdly, vegetation tree species include dominant tree species, understory vegetation, and plant succession. Mostly dominant forests are a mixture of coniferous and deciduous trees, with commonly occurring in Scots pine (*Pinus sylvestris*), birch (*Betula* spp.), and aspen (*Populus tremula*) trees. Similarly, understory vegetation composed of a variety of shrubs, ferns, and mosses with a dense canopy of the trees creates a shaded environment that supports shade-tolerant species (Wei et al., 2024).

### 7.2 Seminatural habitats

Its observed that BFT is renowned for survival in harsh environmental conditions, and these conditions likely occur in seminatural habitats where they have most of their processes and biodiversity intact (Mohy-Ud-Din et al., 2024). Seminatural grasslands include mesic meadows, dry grasslands, heathlands, woodlands, wetlands, and marches and ultramafic soils (Casavecchia et al., 2021). Mesic meadows are seminatural open habitats with high biodiversity where BFT is one of the common species found in these meadows, which are often conserved through traditional agricultural practices such as mowing (Bretzel et al., 2024). In dry grasslands, it survives in water-stress conditions with low moisture content. That leads to less availability of nutrients and shifting of plants towards nitrogen fixation (Bretzel et al., 2024). Moreover, seminatural grassland areas are woodland edges and clearings, which are famous for receiving abundant sunlight and allowing the growth of a variety of plant species, including BFT (Green, 2024). In wetlands and marshes, BFT is commonly found due to high moisture levels and nutrient-rich soil, which allows the plant’s adaptability in these varied conditions (Wang et al., 2025). Low nutrient availability drives plants to form symbiotic associations with rhizobia. Ultramafic soils, characterized by toxic metals and low macronutrient availability, further reinforce this shift. Under such conditions, plants rely on rhizobia symbiosis to fix atmospheric nitrogen and compensate for nutrient limitations (Vincent et al., 2022).

Seminatural grasslands are different from agricultural lands that need soil management, biodiversity conservation, and habitat structure management. On the other hand, urban areas suffered from pollution, habitat, and human disruption (Shipley et al., 2024). In agricultural land management involves cultural practices such as plowing, fertilization, and pesticide application and these alterations affect soil nutrient availability to BFT but on the other hand seminatural habitats have more stable soil conditions with less human intervention (Fedrizzi, 2023) Secondly, there is a risk of losing biodiversity of BFT populations while seminatural habitats support a wider range of complex and resilient ecosystems. So, it’s suitable for wild BFT population conservation (Shipley et al., 2024). Thirdly, habitat structure contains uniform fields with few natural features, while on the other hand, seminatural habitats have a more varied structure with different plant heights, densities, and types (Litovska et al., 2025). Similarly, urban areas are highly fragmented with buildings, roads, and other infrastructure, and can isolate their populations and limit their ability to spread and reproduce (Litovska et al., 2025). Contrarily, seminatural habitats are constantly allowing for better connectivity and gene flow among plant populations (Shipley et al., 2024). Pollution affects levels of air, soil, and water pollution in urban areas, which can adversely influence the growth and survival of BFT. But seminatural habitats provide a healthier environment for the plant (Shipley et al., 2024). Human disturbance refers to trampling, construction, and landscaping, and these actions can harm the habitats. While semi-natural habitats experience less direct disturbance, allowing the plant to thrive (Shipley et al., 2024).

### 7.3 Role of BFT in ecosystem function

BFT is important to the ecosystem due to various interactions with pollinators, nitrogen fixation and other species benefits (Jach et al., 2022). The bees, butterflies and other insects that are attracted to this species, bright yellow flowers pollinate them because they are the main key to the reproduction of plants. The degree of urbanization may have a significant effect on the pollinator community and success of pollination by BFT (Morris et al., 2021). For instance, at the local scale, species richness and pollinator abundance increase when the number of plants and the size of seminatural areas have an impact (Morris et al., 2021). Similarly, plants relying solely on symbiotic nitrogen fixation have lower biomass, but higher reproductive output compared to those receiving nitrogen fertilization. Furthermore, it can tolerate and fix nitrogen even under metal stress conditions like nickel, cobalt, and chromium (Jach et al., 2022). Besides its role in nitrogen fixation and pollination, BFT provides support for other species in the ecosystem, and its dense growth can create a microhabitat that shelters various insects and small animals. It also serves as a food source for herbivores; however, the plant’s chemical defenses, such as tannins and proanthocyanidins, can influence herbivory (Morris et al., 2021). For instance, the presence of tannins can prevent certain herbivores, while others may be adapted to consume the plant despite these threats. This balance between providing food resources and protecting against herbivores is essential for maintaining biodiversity and ecosystem stability (Griffin, 2022).

### 7.4 Major threats to conservation status and genetic diversity (habitat fragmentation, management degradation, climate extremes)

The genetic diversity of BFT in the Hemi-boreal zone is threatened by habitat fragmentation, degradation of traditional management practices and increasing climate extremes. Fragmentation resulting from agricultural intensification, urban expansion and infrastructure development restricts gene flow among populations. Management degradation, including abandonment or over-intensification of grasslands, alters species composition and reduces population size and viability. Climate extremes such as droughts, heat waves and increasing temperature variability impose additional stress on populations, potentially leading to genetic erosion and local declines.

### 7.5 Analysis of existing conservation strategies and gaps

Conservation strategies for BFT include the identification of genetically important populations, *in-situ* and *ex-situ* conservation, genetic monitoring, habitat management and restoration, and research collaboration (Chen et al., 2023). *In-situ* conservation focuses on protecting seminatural habitats and managing protected areas that contain populations with distinct genetic backgrounds and ecotypes (Wippel et al., 2021; Zhang et al., 2023). Sustainable grazing practices are essential, as overgrazing reduces population abundance and genetic diversity, whereas appropriate grazing intensity promotes species persistence and genetic variation (Abraham et al., 2015). Controlling invasive species is also necessary to reduce competitive pressure and maintain ecological balance (White et al., 2025). *Ex-situ* conservation involves the development of seed banks and germplasm collections to preserve genetic material from diverse populations for future restoration and breeding programs (Katoch, 2022; Leger et al., 2024; McLean-Rodríguez et al., 2021; Nadarajan et al., 2023). Regular genetic monitoring using molecular markers allows assessment of population structure, detection of inbreeding and identification of genetic differentiation within and among populations (Abraham et al., 2015; Hoban et al., 2022). Despite these efforts, gaps remain in long-term monitoring, integration of genetic data into restoration planning and coordinated conservation action across regions.

## 8 Knowledge gaps and future research directions

Significant knowledge gaps exist regarding the genetic diversity of BFT across different habitat types, its symbiotic rhizobia partners in northern temperate regions, pollen thermotolerance and fine-scale genomic structure in the Hemi-boreal zone of the Baltic States (Abraham et al., 2015; Ampomah and Huss-Danell, 2011; Chen et al., 2023). Most research has focused on agronomically important legumes such as *Vicia*, *Trifolium* and *Pisum*, while comparatively little attention has been given to the *Lotus* genus (Ampomah and Huss-Danell, 2011). Recent global genomic variation maps of BFT highlight the need for region-specific studies addressing population structure and molecular adaptation in the Baltic Hemi-boreal context (Chen et al., 2023).

Future research should include genetic and genomic analyses of rhizobia symbionts using whole genome sequencing and metagenomics to better understand symbiotic diversity and nitrogen fixation efficiency. Habitat-specific population genetic studies across forests, meadows and wetlands are needed to identify adaptive genetic variants and prevent genetic erosion. Investigation of pollen thermotolerance mechanisms, including the role of heat shock proteins and antioxidant pathways, would clarify reproductive resilience under climate change. Population genomics, resequencing regional accessions and association studies targeting stress tolerance, growth and forage quality will support conservation and breeding programs and improve management of genetic resources in the Baltic States.

## 9 Conclusion

The genetic diversity of BFT in the Hemi-boreal zone of the Baltic States constitutes a cornerstone of ecological sustainability, agricultural resilience, and biodiversity conservation. As demonstrated in this review, BFT exhibits unique adaptive traits that enable it to thrive in unstable and marginal environments of northern Europe. Its ability to grow in nutrient-poor soils, tolerate abiotic stresses, and form efficient symbioses with nitrogen-fixing bacteria makes it valuable both as a forage species and as an ecological restorer. The species’ widespread occurrence in Lithuania, Latvia, and Estonia further reveals a largely unexplored yet substantial reservoir of genetic diversity with relevance to local and continental sustainability objectives. Ecological, genetic, and agronomic evidence indicates that BFT contributes to biodiversity conservation in natural grasslands while supporting low-input agricultural systems. However, the rapid decline of seminatural meadows and pastures underscores the urgent need to conserve native legume genetic resources. Hemi-boreal ecotypes provide a critical genetic buffer against habitat degradation, climate warming, and increasing anthropogenic pressures. Molecular marker analyses have revealed extensive genetic variation within and among populations, forming a foundation for marker-assisted selection and breeding programs. Climatic variability, soil heterogeneity, and biotic interactions drive fine-scale genetic differentiation across the Baltic region, reflecting the species’ high adaptive capacity. Preserving locally adapted populations is therefore both an ecological imperative and a strategic policy measure for sustainable forage based agriculture. Molecular tools ranging from ISSR markers to SNP-based platforms are essential for germplasm conservation and the development of ecotype specific cultivars. Integrating ecological knowledge with high-resolution molecular data enables the development of multifunctional cultivars that balance productivity and resilience. Moreover, the ecological plasticity of BFT enhances mixed-species pastures by sustaining pollinator networks, improving soil fertility, and increasing system resilience. In conclusion, the integration of molecular understanding with ecological application highlights BFT as a key species for mitigating biodiversity loss, adapting to climate change, and promoting long-term agricultural sustainability.
